# MCPIP1 functions as a safeguard of early embryonic development

**DOI:** 10.1038/s41598-023-44294-1

**Published:** 2023-10-07

**Authors:** Agata Lichawska-Cieslar, Weronika Szukala, Tomasz K. Prajsnar, Niedharsan Pooranachandran, Maria Kulecka, Michalina Dabrowska, Michal Mikula, Krzysztof Rakus, Magdalena Chadzinska, Jolanta Jura

**Affiliations:** 1 Department of General Biochemistry, Faculty of Biochemistry, Biophysics and Biotechnology, Jagiellonian University, Gronostajowa 7, 30-387 Kraków, Poland; 2 Doctoral School of Exact and Natural Sciences, Jagiellonian University, Lojasiewicza 11, 30-348 Kraków, Poland; 3 Department of Evolutionary Immunology, Institute of Zoology and Biomedical Research, Faculty of Biology, Jagiellonian University, Gronostajowa 9, 30-387 Kraków, Poland; 4 Medical Center for Postgraduate Education, Department of Gastroenterology, Hepatology and Clinical Oncology, Marymoncka 99/103, 01-813 Warsaw, Poland; 5 Maria Sklodowska-Curie National Research Institute of Oncology, Roentgena 5, 02-781 Warsaw, Poland

**Keywords:** Embryogenesis, RNA

## Abstract

Monocyte chemoattractant protein-induced protein 1 (MCPIP1), also called Regnase-1, is an RNase that has been described as a key negative modulator of inflammation. MCPIP1 also controls numerous tumor-related processes, such as proliferation, apoptosis and differentiation. In this study, we utilized a zebrafish model to investigate the role of Mcpip1 during embryogenic development. Our results demonstrated that during embryogenesis, the expression of the *zc3h12a* gene encoding Mcpip1 undergoes dynamic changes. Its transcript levels gradually increase from the 2-cell stage to the spherical stage and then decrease rapidly. We further found that ectopic overexpression of wild-type Mcpip1 but not the catalytically inactive mutant form resulted in an embryonic lethal phenotype in zebrafish embryos (24 hpf). At the molecular level, transcriptomic profiling revealed extensive changes in the expression of genes encoding proteins important in the endoplasmic reticulum stress response and in protein folding as well as involved in the formation of primary germ layer, mesendoderm and endoderm development, heart morphogenesis and cell migration. Altogether, our results demonstrate that the expression of *zc3h12a* must be tightly controlled during the first cell divisions of zebrafish embryos and that a rapid decrease in its mRNA expression is an important factor promoting proper embryo development.

## Introduction

Implantation of the blastocyst is necessary for embryonic development to occur. In mammals, uterine implantation is associated with numerous structural and molecular changes in the luminal epithelia^1^. Interestingly, this process is closely correlated with the inflammatory mechanism, where during the attachment of blastocysts to the uterus, modulations in the expression of different growth factors, chemokines and cytokines, such as TNF, IL-6 and prostaglandin E2 (PGE2), are observed, as well as vascularization and infiltration of immune cells from the blood to the endometrial tissue^2–5^. The inflammatory process is essential for implantation, but during the next stage of pregnancy, anti-inflammatory factors are induced to prevent rejection of the fetus. Thus, proper embryonic development is strictly dependent on optimal microenvironmental conditions. This microenvironment must provide optimal temperature, oxygen tension, pH, and nutrients to ensure embryo survival. Stress during the embryonic period may impair fetal development and result in embryo mortality^6,7^.

In human, monocyte chemoattractant protein-induced protein 1 (MCPIP1), also known as Regnase-1 and encoded by the *ZC3H12A* gene, possesses a PilT N-terminus domain (PIN) that exerts RNAse properties. It has been shown that MCPIP1 degrades transcripts coding for mediators of inflammation: IL-1β^8^, IL-6^9^, IL-12p40^10^ and IL-2^11^ and its own transcript^10^. Further studies have indicated that MCPIP1 also plays an important role in the suppression of miRNA activity and biogenesis via cleavage of the terminal loops of precursor miRNAs (pre-miRNAs), counteracting Dicer, a key ribonuclease in miRNA processing^12^. MCPIP1 transcript expression is induced by Toll-like receptor (TLR) ligands^9,13^ and proinflammatory cytokines, such as IL-1β and TNFα^8^.

In vivo studies have shown that MCPIP1 plays a critical role in preventing autoimmune conditions. *Zc3h12a* knockout mice display severe anemia, augmented serum immunoglobulin levels and autoantibody production. In macrophages of *Zc3h12a*^-/-^ mice, elevated expression levels of IL-6, IL-12p40 and IL-1β were observed^9,14^. Changes in the immune response of knockout mice are not only the result of MCPIP1 involvement in mRNA decay and miRNA biogenesis but also of its influence on the activity of some transcription factors. To date, MCPIP1 has been confirmed to negatively regulate the activity of NF-ĸB and AP1, which is essential in the regulation of the synthesis of mediators controlling inflammatory and immune responses^14–17^.

In addition, evidence indicates that MCPIP1 is involved in cell cycle arrest^18^, apoptosis^19^ and regulation of cell differentiation^20–22^. Thus, we hypothesized that MCPIP1 might safeguard early development and regulate crucial processes in the early stages of embryonic development, such as cell differentiation, cell division, apoptosis, angiogenesis, and regulation of inflammatory processes. A zebrafish (*Danio rerio*) model was utilized, which is a powerful vertebrate platform widely used to investigate developmental processes. First, we investigated whether the temporal expression pattern of the *zc3h12a* transcript, encoding Mcpip1, is changed during embryogenesis. Subsequently, we determined how the modulation of Mcpip1 levels affects the early development of zebrafish.

## Results

### The zebrafish genome contains four unique members of the PIN domain-like superfamily

**Figure 1:**
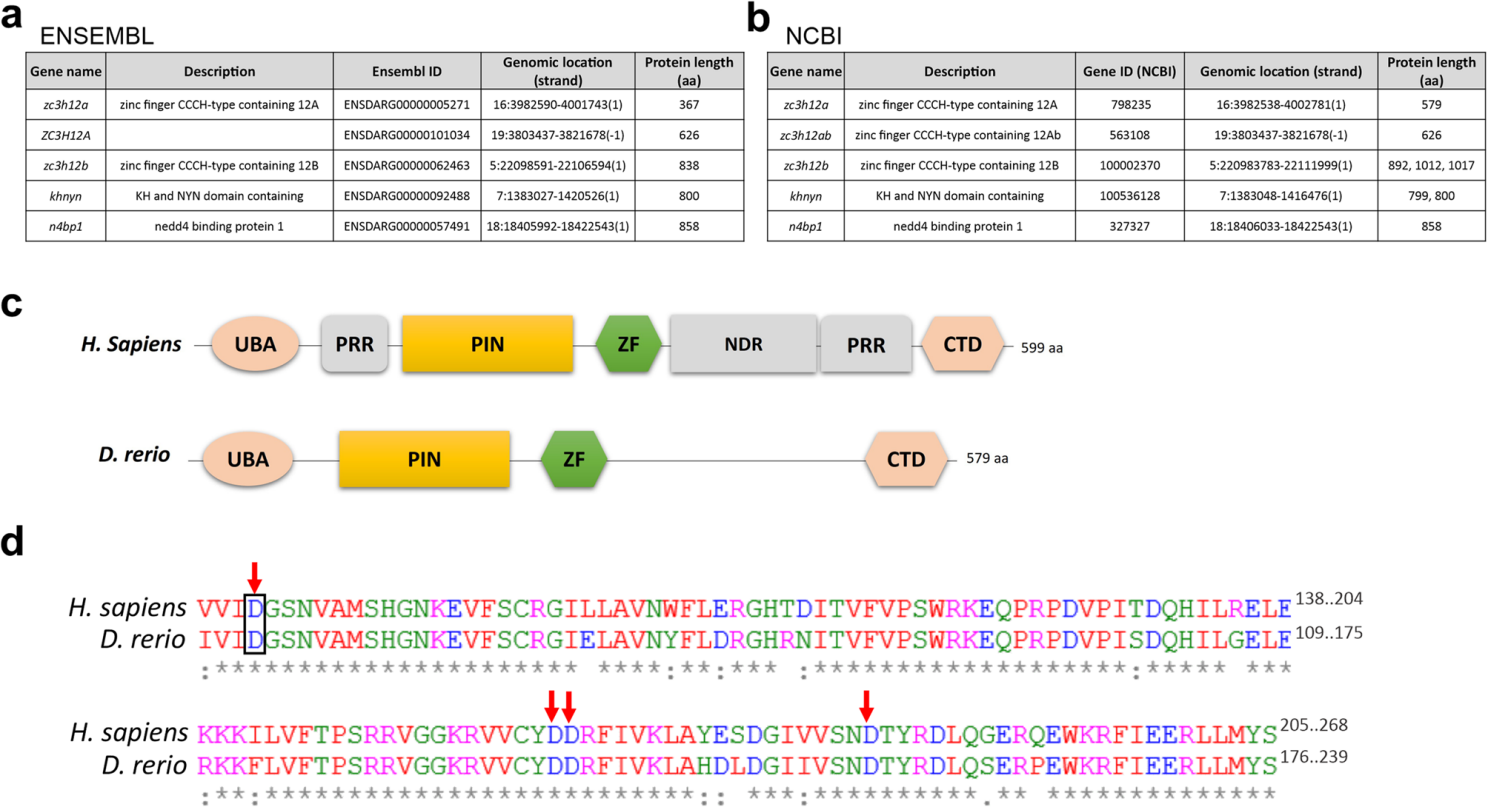
The zebrafish ortholog of human MCPIP1 shares a similar domain structure. (**a**) List of genes containing the *Zc3h12a*-like NYN domain in the zebrafish genome based on the Ensembl database. (**b**) List of genes containing the *Zc3h12a*-like NYN domain in the zebrafish genome based on the NCBI database. (**c**) Domain comparison of human MCPIP1 and its zebrafish ortholog Mcpip1. UBA—ubiquitin-associated domain, PRR—proline-rich region, PIN—PilT N-terminus nuclease domain, ZF—zinc finger motif, NDR—disordered region, CTD—C-terminal conserved domain. (**d**) Alignment of the amino acid sequences of human and zebrafish PIN domains. Asterisk (*) denotes an identical residue, a colon (:) denotes conserved substitutions, and a period (.) denotes semiconserved substitutions. The arrows indicate four aspartic acid residues, which are critical for Mcpip1/MCPIP1 catalytic activity. Black rectangle indicates position of the D112N mutation within the PIN domain of zebrafish Mcpip1.

To begin investigating the potential function of the Mcpip1 RNase in zebrafish, we performed bioinformatic searches and alignments. According to the Ensembl and NCBI databases, the zebrafish genome contains five members of the PIN domain-like superfamily (Fig. 1a,b), also recently showed by Yang *et al*.^23^. The full-length zebrafish *zc3h12a* sequence (XM_021466808.1; Gene ID: 798235; NCBI) encodes a 579 amino acid Mcpip1 protein with high (82.90%) amino acid identity to common carp Mcpip1 and intermediate (~ 50%) amino acid identity to amphibian, reptilian, bird and mammalian MCPIP1. The maximum likelihood (ML) phylogenetic tree for MCPIP1 proteins (Supplementary Fig. S1) demonstrates that the fish species cluster together and form a clade separate from the nonfish species. However, a direct comparison of the zebrafish orthologs of the human PIN-domain like-superfamily members indicated that zebrafish and human MCPIP1 proteins possess a similar domain structure and 87.79% identity within the PIN domain (Fig. 1c, Supplementary Fig. S2). The most highly conserved region is the PIN domain, the catalytic center of Mcpip1 (Fig. 1d).

### The zebrafish ortholog of *ZC3H12A* is downregulated during early embryonic development

**Figure 2:**
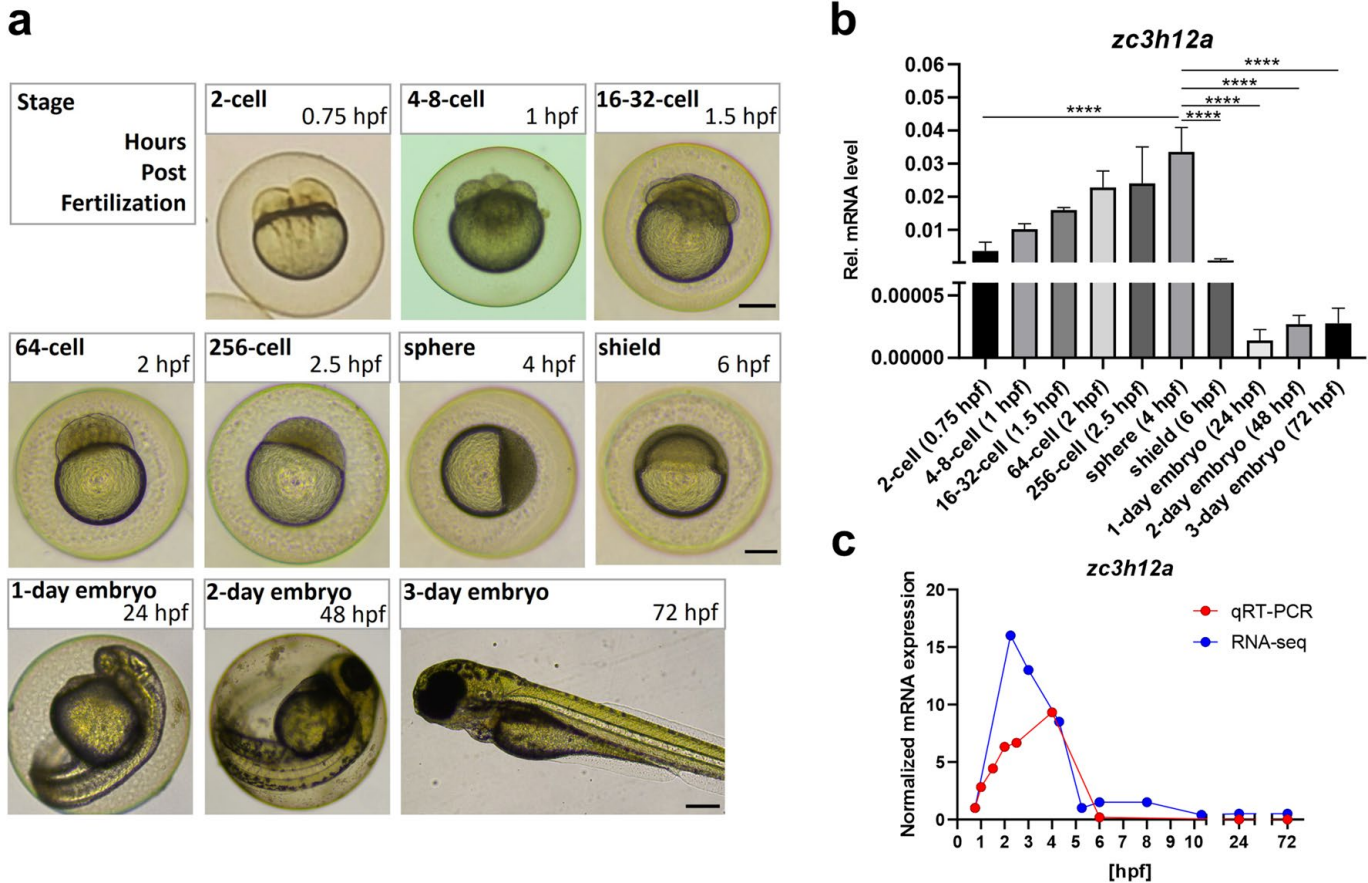
The expression of *zc3h12a* is tightly controlled during early embryonic development in zebrafish. (**a**) One-cell fertilized eggs were incubated under standard conditions and collected at the indicated timepoints for RNA analysis. Representative pictures of zebrafish embryos at various developmental stages are shown. (**b**) Real-time PCR analysis of the *zc3h12a* expression level at different developmental stages of zebrafish. (**c**) Graph represents the level of *zc3h12a* mRNA based on the RNA-Seq data (PMID: 29,144,233). For comparison of both datasets, mean values of *zc3h12a* mRNA levels from qRT-PCR were also shown (normalized values of data presented on Fig. 2a). In both cases the level of *zc3h12a* mRNA was normalized to the expression level at 2-cell stage (0.75 hpf). hpf—hours post fertilization *n* = *3*. *Rps11* was used as a reference gene. Data represent the mean ± SD, ****p < 0.0001 by one-way ANOVA (only comparisons with *p* < 0.0001 are shown). Scale bar = approx. 250 µm.

To investigate the temporal expression pattern of the zebrafish *zc3h12a* gene during early embryonic development, quantitative real-time PCR (qRT‒PCR) was performed on whole embryo-derived RNA. One-cell stage fertilized zebrafish eggs were collected and incubated at a standard temperature of 28 °C as described in the methods. The embryos were monitored for cell division and collected at different developmental periods over 72 h. As expected, the first divisions were observed synchronously at ~ 20-min intervals (Fig. 2a)^24^. For the isolation of RNA, ~ 10 embryos were pooled at each timepoint.

We found that the *zc3h12a* gene follows dynamic changes in expression during early zebrafish development. The level of *zc3h12a* mRNA gradually increases during the first embryonic divisions, peaking at the sphere stage (4 h post fertilization [hpf]) and then significantly rapidly declining during transition into the shield stage and 1-day embryo (Fig. 2b and Supplementary Fig. S3a). In addition, we analyzed the global transcriptomic RNASeq data by White et al*.*^25^, which provides information about gene expression changes during early zebrafish embryogenesis. Both approaches showed a similar trend of *zc3h12a* gene expression fluctuations during zebrafish embryogenesis (Fig. 2c).

### Transient thermal shock during early zebrafish development enhances the expression of zc3h12a mRNA

**Figure 3:**
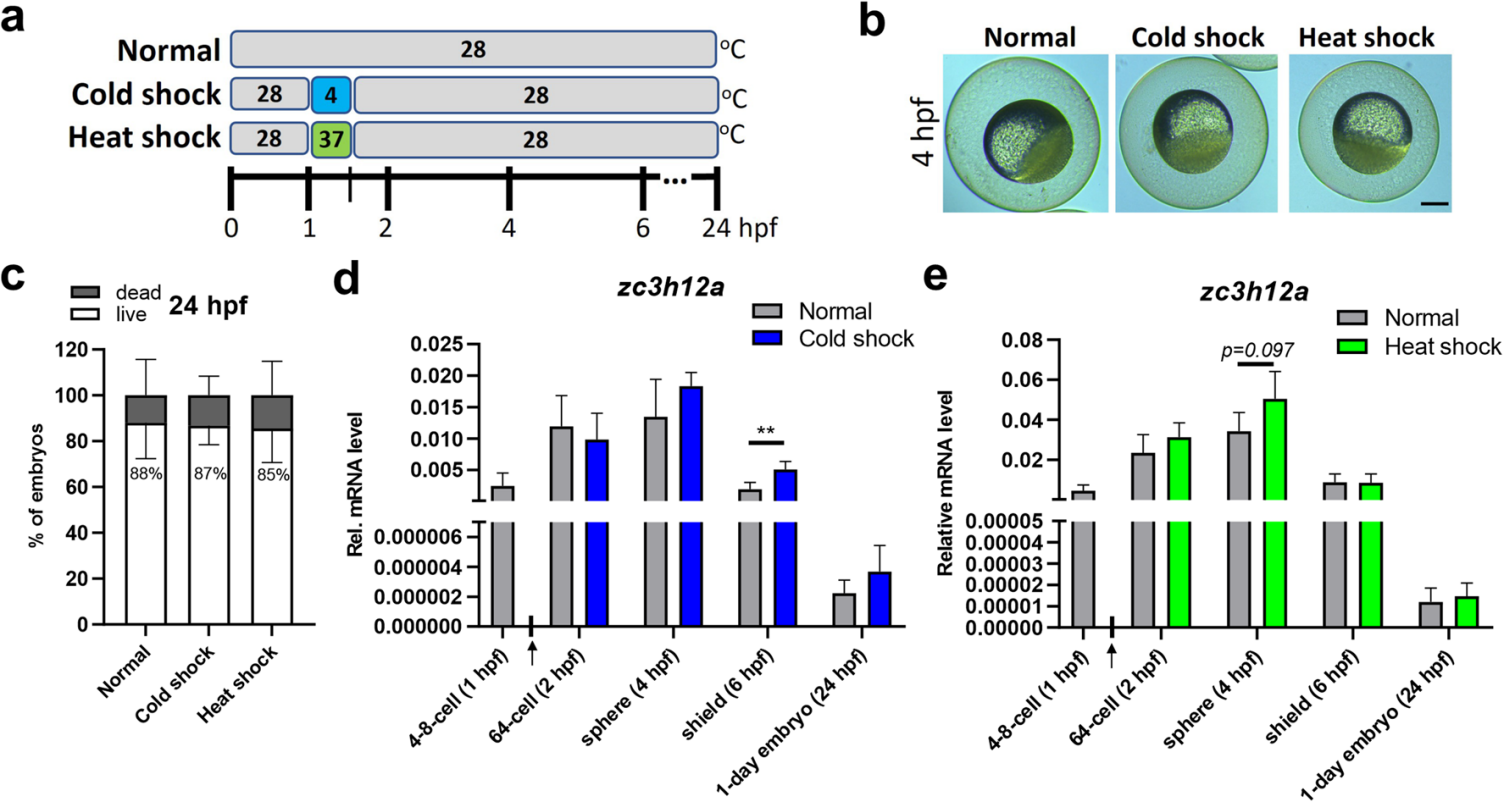
Effect of transient thermal shock on the expression of zebrafish *zc3h12a* during early embryonic development. (**a**) Zebrafish embryos that were incubated at standard 28 °C temperature for 1 h were subjected to a transient 30-min heat shock (37 °C) or cold shock (4 °C) as indicated on the schematic diagram. Embryos were collected for RNA analysis at the indicated timepoint. (**b**) Representative pictures of zebrafish embryos at 4 hpf. **c.** Percentages of live and dead embryos at 24 hpf. (**d**,**e**) Real-time PCR analysis of the *zc3h12a* expression level in zebrafish embryos subjected to each thermal shock. The arrow on the X-axis indicates the onset of the 30 min thermal shock. hpf—hours post fertilization. *n* = *4*. *Rps11* was used as a reference gene. Data represent the mean ± SD, **p < 0.01 by an unpaired *t*-test. Scale bar = approx. 250 µm.

We next sought to investigate whether an external shock during early development affects the expression of the *zc3h12a* gene. The thermal stimulation approach was performed, due to the fact that the more commonly used zebrafish infection/inflammation models could not be efficiently utilized to monitor processes in early embryos (0–24 hpf)^26^. Synchronous populations of 2–4 cell embryos that had been cultured at 28 °C were subjected to a 30-min thermal shock at 4 °C (cold shock) or 37 °C (heat shock). Control embryos were kept at a standard temperature of 28 °C. After a transient temperature shift, the embryos were incubated at the standard 28 °C and collected at the indicated timepoints for RNA analysis **(**Fig. 3a**)**.

We ensured that the developmental stages of embryos subjected to cold or heat shock followed similar timing as the control siblings **(**Fig. 3b**)** so the analysis of *zc3h12a* gene expression was not biased by a potential shift of developmental stage. Transient thermal shocks did not impair the overall embryonic survival rates at the 24-h stage **(**Fig. 3c**)**.

Quantitative RT‒PCR analysis revealed that the expression of *zc3h12a* was significantly enhanced in response to the 30 min cold shock, reaching levels ~ 2.5-fold higher than those in the control embryos at the shield stage (6 hpf; Fig. 3d). The 37 °C shock suggested a similar tendency on the *zc3h12a* mRNA expression level at the sphere stage (4 hpf), with a ~ 1.5-fold increase (with adj. *p value* 0,097; Fig. 3e).

### Overexpression of *zc3h12a* impairs early embryonic development in zebrafish

**Figure 4:**
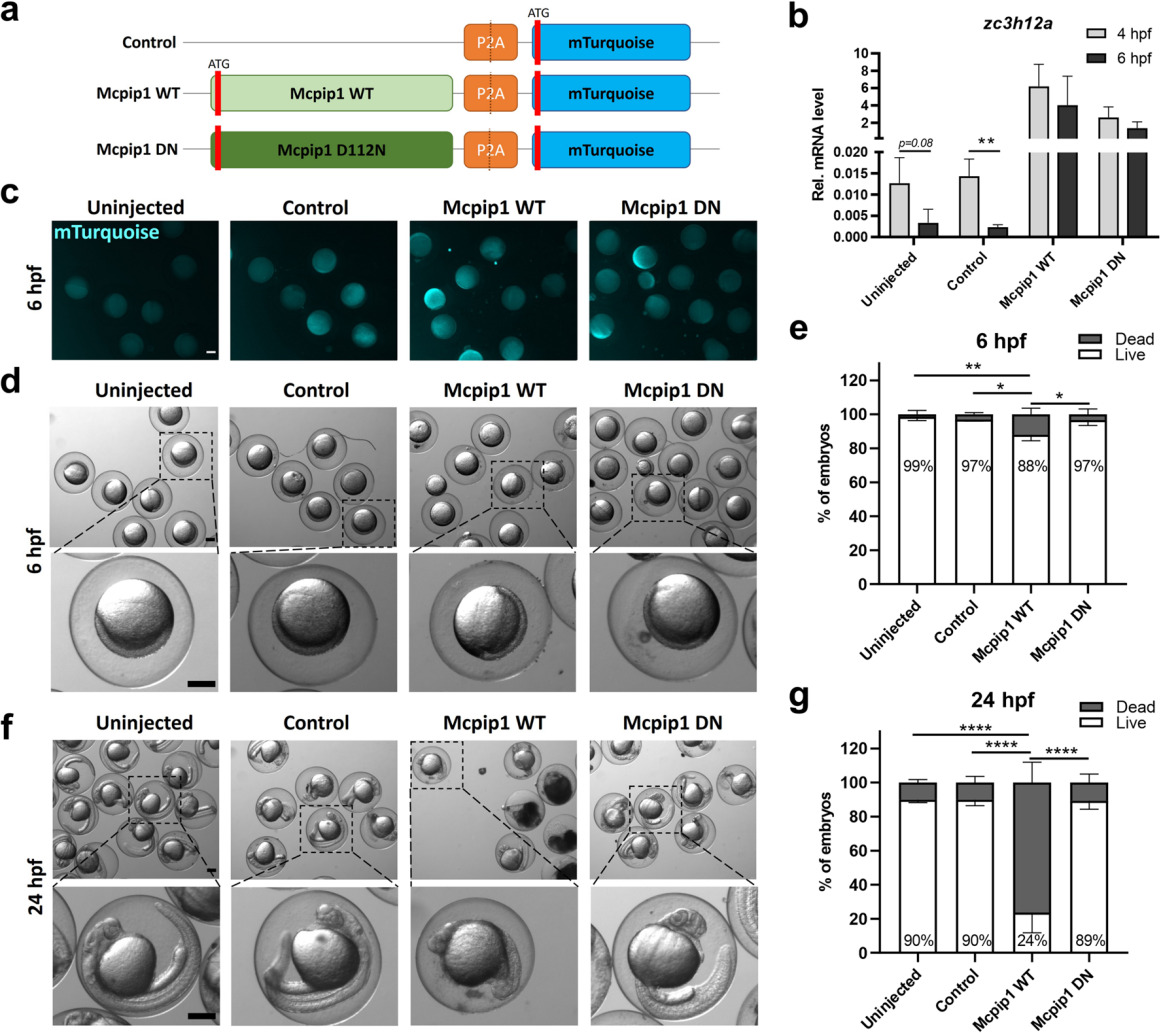
Overexpression of catalytically active Mcpip1 impairs early development in zebrafish. (**a**) Schematic diagram of control and Mcpip1 overexpression constructs. The sequences were cloned into the pCS2 expression vector. (**b**) Real-time PCR analysis of *zc3h12a* expression levels in uninjected and microinjected control, Mcpip1 WT or Mcpip1 DN mRNA zebrafish embryos at 4 hpf and 6 hpf. *n* = *3*. (**c**) Representative fluorescence (mTurquoise) images of embryos at 6 hpf. (**d**) Representative bright field images of embryos at 6 hpf. (**e**) Percentages of live and dead embryos at 6 hpf. (**f**) Representative bright field images of embryos at 24 hpf. (**g**) Graph indicating the percentages of live and dead embryos at 24 hpf. *n* = *3*. *Rps11* was used as a reference gene. Data represent the mean ± SD, *p < 0.05, **p < 0.01, ****p < 0.0001 by an unpaired *t*-test **(b)** or one-way ANOVA **(d,f)**. Scale bar = approx. 250 µm.

Zebrafish Mcpip1 was overexpressed by microinjection of in vitro transcribed mRNA encoding the zebrafish Mcpip1-P2A-mTurquoise protein into the yolks of one-cell stage embryos. Expression of Mcpip1 was tied to mTurquoise expression via self-cleaving P2A peptide. This strategy enabled visualization of exogenously expressed protein. For overexpression of catalytically inactive Mcpip1, one of the four conserved aspartic acid residues within the RNase domain **(**Fig. 1d**)** was substituted with asparagine (Mcpip1-D112N, herein Mcpip1 DN). It has previously been shown that a single mutation in the catalytic center of Mcpip1 is sufficient to completely abolish its RNase activity^9^. As a control, a construct encoding only P2A-mTurquoise was used **(**Fig. 4a**)**. Prior to microinjection, agarose electrophoresis was performed, which indicated high quality of in vitro transcribed mRNAs **(**see Supplementary Fig. S3b).

Embryos were sampled at 4, 6 and 24 h after microinjection. Exogenous *zc3h12a* mRNA was clearly detected at the sphere (4 hpf) and shield (6 hpf) stages. We also found that the microinjection procedure itself did not affect the kinetics of the *zc3h12a* mRNA expression pattern at those two developmental stages **(**Fig. 4b**)**.

We further confirmed that mTurquoise expressed from microinjected mRNA was detected in all modified embryos at the shield stage (6 hpf; Fig. 4c**)**. We also observed that at this stage, overexpression of wild-type (WT) but not the RNase-dead mutant did not affect embryo morphology (Fig. 4d). However, embryonic lethality increased by ~ 10% **(**Fig. 4e**)**. Consistent with this finding, 24 h after microinjection, most Mcpip1-overexpressing embryos showed gross morphological abnormalities; thus, the overall lethality rate was significantly higher than that of the control embryos **(**Fig. 4f–g**)**.

### Increased activity of Mcpip1 leads to profound transcriptomic changes in developing zebrafish embryos

**Figure 5:**
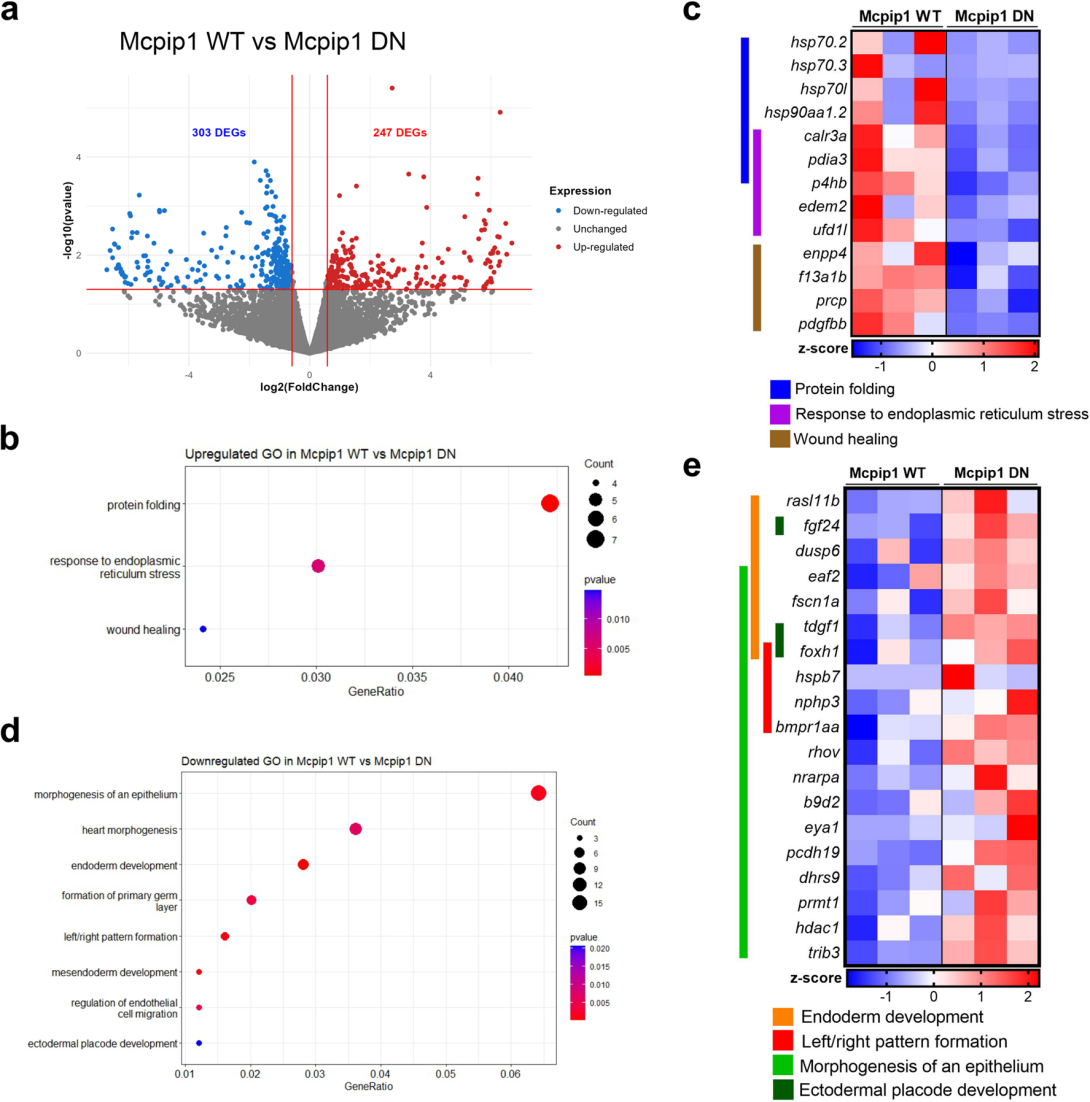
Transcriptome analysis of zebrafish embryos overexpressing Mcpip1. (**a**) Volcano plot for the RNA-Seq dataset indicating differentially expressed genes with *p value* < 0.05 and *fold change (FC)* > 1.5 between embryos microinjected with Mcpip1 WT and Mcpip1 DN mRNA (6 hpf). *n* = *3*. (**b**) Gene Ontology (GO) enrichment analysis of upregulated biological processes in Mcpip1 WT. (**c**) Heatmap illustrating the expression levels of selected upregulated DEGs. (**d**) Gene Ontology (GO) enrichment analysis of downregulated biological processes in Mcpip1 WT. (**e**) Heatmap illustrating the expression levels of selected downregulated DEGs.

Transcriptomic profiling was performed on RNA isolated from viable Mcpip1 WT and Mcpip1 DN-overexpressing embryos collected at the shield stage (n = 3 of ~ 10 pooled embryos per condition). Differentially expressed genes (DEGs) were defined with a threshold of *p* value < 0.05 and fold change > 1.5. Accordingly, the expressions of 247 genes were significantly upregulated and those of 303 genes were downregulated in zebrafish embryos overexpressing WT Mcpip1 compared to those overexpressing the Mcpip1 DN mutant **(**Fig. 5a**)**.

Gene Ontology (GO) enrichment analyses revealed that upregulated DEGs were significantly enriched in biological processes (BP) mostly related to the response to protein folding and endoplasmic reticulum stress, examples of which are *calr3a* (*calreticulin 3a*), *edem 2* (*ER degradation enhancer*) and *hsp70* (*heat shock protein 70*) family genes **(**Fig. 5b,c**)**.

In contrast, the downregulated DEGs were enriched in pathways related to endoderm development, left/right pattern formation, organ morphogenesis and formation of the primary germ layer and ectodermal placode development **(**Fig. 5d,e**)**.

### Oscillation of *zc3h12a* expression during early embryonic development in zebrafish inversely correlates with changes in *nrarpa* and *rasl11b* expression levels

**Figure 6:**
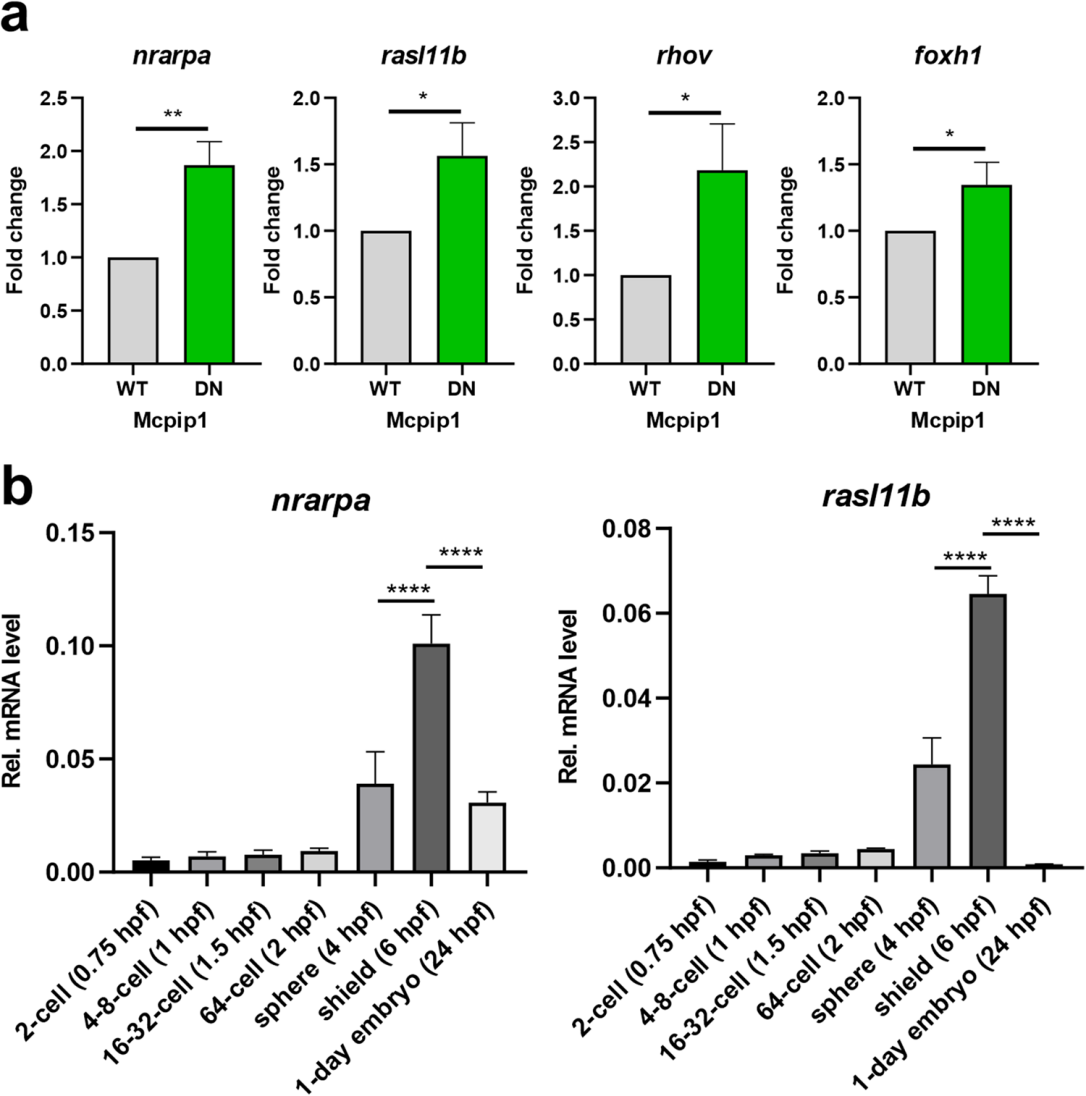
Expression pattern of *nrarpa*, *rasl11b, rhov* and *foxh1* genes during zebrafish embryogenesis. (**a**) Real-time PCR analysis of *nrarpa*, *rasl11b*, *rhov* and *foxh1* expression levels in zebrafish embryos overexpressing Mcpip1 WT or Mcpip1 DN (6 hpf). (**b**) Real-time PCR analysis of *nrarpa* and *rasl11b* expression levels during early embryonic development in zebrafish. *n* = *3*. *Rps11* was used as a reference gene. Data represent the mean ± SD, *p < 0.05, **p < 0.01, ****p < 0.0001 by an unpaired *t*-test (**a**) or one-way ANOVA (**b**). For (**b**) only selected developmental stages have been compared.

Based on our RNASeq analysis, we next selected four significantly downregulated DEGs in zebrafish embryos overexpressing WT Mcpip1: *nrarpa* (*Notch-regulated ankyrin repeat-containing protein A*), *rasl11b* (*RAS-like family 11 member B*), *rhov* (*ras homolog family member V*) and *foxh1* (*forkhead box H1*)*.* These DEGs were functionally assigned to the most significantly downregulated processes **(**Fig. 5d,e**),** and their expression levels were validated by qRT‒PCR (Fig. 6a).

In the next step, the expression patterns of two transcripts, *nrarpa* and *rasl11b,* were analyzed over the course of zebrafish embryogenesis by qRT‒PCR **(**Fig. 2a**)**. We found that their expression levels undergo extensive changes during the transition from 64-cell to 1-day embryos **(**Fig. 6b**)**. Moreover, the relative expression levels of those genes peaked at the shield stage, which correlates with the time at which *zc3h12a* expression profoundly decreases **(**Fig. 2b**)**.

## Discussion

Zebrafish (*D. rerio*) is a leading model for studying developmental biology because the genome is known, fertilization occurs externally, and development is very fast. At 48 h after fertilization, zebrafish embryos form complete organ systems, including the heart, intestine and blood vessels. In addition, embryos are transparent and develop outside the uterus, making it even easier to track developmental stages^24,27^.

MCPIP1 expression is inducible, and mRNA levels change rapidly after stimuli associated with cell differentiation, stress induction or pathogen infection^8,9,28^. Consequently, an increase in MCPIP1 is correlated with a decrease in the levels of many transcripts encoding regulators of inflammation, regulators of cell differentiation and division, and regulators of apoptosis and angiogenesis. Here, we wanted to test the role of MCPIP1 in embryological development. Comparison of the amino acid sequences showed differences between human MCPIP1 and its zebrafish ortholog. However, the PIN domain has high homology with the conserved codons encoding four acidic amino acid residues that form the putative active site, which is essential for the ribonucleolytic activity of MCPIP1.

We found that the expression of *zc3h12a* is tightly controlled within the first cell divisions of zebrafish. Mcpip1 mRNA levels increase from the 2-cell stage up to the sphere stage (6 h after fertilization); later, its level drops dramatically. Thus, starting in the sphere stage, Mcpip1-dependent transcript level regulation is silenced. Relatively high expression of Mcpip1 during first hours post fertilization may suggest potential role of this RNase during the decay of maternal mRNA (which in zebrafish is completed by 6 hpf^29^), but this hypothesis requires further investigation.

MCPIP1 is encoded by an inducible gene that has the ability to rapidly respond to microenvironmental changes and responds quickly to various kinds of stress^8,9,28,30,31^. In this study, mild external stress was induced by a temporary change in temperature, which led to transient modulations of *zc3h12a* gene expression, with consequent effects on the RNA profile. To determine what consequences an increase in Mcpip1 transcript levels might have on embryonic development, we used zebrafish models overexpressing WT Mcpip1 or its mutant form containing a mutation in the active site of the PIN domain.

We found that ectopic expression of WT Mcpip1 resulted in a 10% death rate of zebrafish embryos at 6 h post-fertilization and almost 90% after 24 h, while the model expressing the inactive form of Mcpip1 resembled control models (uninjected zebrafish embryos or injected with an empty vector). These results provide evidence for a significant effect of Mcpip1 on the zebrafish embryo transcriptome. There are many studies in the literature showing that Mcpip1 regulates genes involved in apoptotic processes^16,32–34^, so high embryonic lethality may be a consequence of the dysregulation of transcripts involved in this process. In our model, activation of the endoplasmic reticulum (ER) stress response pathway observed in the Mcpip1-overexpressing embryos already at 6 hpf **(**Fig. 5c**)** could have provoked the activation of apoptotic cell death, as prolonged ER-stress is a common trigger of apoptosis^35^. Furthermore, Mcpip1-overexpressing embryos showed gross morphological abnormalities. Transcriptome analysis revealed expression differences in mRNA classes encoding proteins important for embryonic development. We observed that, among the genes that were activated, the largest group comprised genes encoding proteins important to the endoplasmic reticulum stress response and involved in protein folding. The activation of these genes may be an indirect consequence of the ectopic expression of Mcpip1 and the accumulation of this protein in embryonic zebrafish cells. However, among the many transcripts downregulated and thus potentially directly degraded by Mcpip1 were those involved in the formation of the primary germ layer, mesendoderm, ectoderm and endoderm development, heart morphogenesis and cell migration. Thus, the change in the expression levels of such developmentally important genes may explain why such high lethality was observed when Mcpip1 levels were elevated during early zebrafish development. For validation of RNA-Seq data by qRT-PCR, four significantly downregulated transcripts in zebrafish embryos, *nrarpa* (*Notch-regulated ankyrin repeat-containing protein A*), *rasl11b* (*RAS-like family 11 member B*), *rhov* (*ras homolog family member V*) and *foxh1* (*forkhead box H1*) were selected. We also showed that the levels of *nrarp*a and *rasl11b* inversely correlate with fluctuations in Mcpip1 mRNA levels during early stages of development **(**Fig. 6b**).** We hypothesize that a rapid decline of Mcpip1 expression level observed at shield stage (6 hpf) contributes to a profound upregulation of *nrarpa*, *rasl11b* and possibly other developmentally important target mRNAs at this stage.

NRARP is a component of the Notch signaling pathway and participates in embryonic development in vertebrates by regulating the segmentation of the body axis. The importance of this transcript in the segmentation process has been documented in lower and higher vertebrates^36–38^. The *rasl11b* gene, on the other hand, encodes a small GTPase protein that is highly conserved among vertebrates; it is expressed in mesendodermal cells, and its expression is controlled by the Nodal pathway. Nodal signaling controls the expression of conserved mesendodermal transcription factors^39^. Interestingly, it has been shown that *rasl11b* knockdown induces a specific “curly tail down” phenotype in zebrafish^39^. Similarly, ectopic overexpression of WT Mcpip1 resulted in embryo malformations, including tail malformations **(**Fig. 4e**)**. The third highly-downregulated transcript, *rhov,* is involved in the signal transduction of the Rho pathway, which is essential for the regulation of gastrulation and neurulation, two major developmental processes of early embryogenesis^40,41^. In addition, our analysis identified many other genes whose levels are directly or indirectly dependent on Mcpip1, e.g., *teratocarcinoma-derived growth factor 1* (*tdgf1*), described as an important regulator in the development of the cardiac tube in mouse embryogenesis^42^, and *forkhead box protein H1* (*foxh1*), essential during zebrafish gastrulation and head and dorsal axis formation^43^. Our RNAseq data also showed a decreasing trend of *neurogenin 1 (neurog1)* expression (*p value* = *0.077*), a key factor directing specialization of neuroectoderm^44^ in 6 hpf embryos overexpressing active Mcpip1 (Supplementary Fig. S3c), which additionally proves observed lethal phenotype.

In conclusion, our studies in the zebrafish model showed that the Mcpip1 level is tightly regulated during embryonic development, while even transient stress leads to rapid induction of the *zc3h12a* gene. Consequently, elevated expression of the *zc3h12a* gene leads to abnormalities in zebrafish development as a result of altered levels of transcripts involved in processes important in embryogenesis. It can be speculated that stress leading to induction of the gene encoding Mcpip1/MCPIP1 will have similar consequences on the developing embryo in higher vertebrates, including humans.

## Materials and methods

### Phylogenetics and bioinformatics

To find MCPIP1 orthologs in the zebrafish genome (GRCz11), the Ensembl database was searched for genes containing a *Zc3h12a*-like NYN domain. Sequences were retrieved from the SwissProt, EMBL and GenBank databases using SRS and/or BLAST (Basic Local Alignment Search Tool)^45^. Amino acid sequence alignment was performed using the Clustal Omega program at EMBL-EBI (https://www.ebi.ac.uk/Tools/msa/clustalo). Phylogenetic trees were constructed on the basis of amino acid differences by the maximum likelihood (ML) method with 500 bootstrap replications using Molecular Evolutionary Genetics Analysis (MEGA) version 11^46^. For metadata analysis of *zc3h12a* mRNA expression profile during zebrafish development, the RNA-seq dataset provided by the Busch-Nentwich lab and available at Expression Atlas, was used^25^.

### Zebrafish husbandry

Zebrafish embryos/larvae were obtained by the natural spawning of adult zebrafish (line AB/TL), which were housed in a continuous recirculating closed-system aquarium with a light/dark cycle of 14/10 h at 28 °C. Larvae were incubated in E3 medium at 28 °C according to standard protocols^47^. The Jagiellonian University Zebrafish Core Facility (ZCF) is a licensed breeding and research facility (District Veterinary Inspectorate in Krakow registry; Ministry of Science and Higher Education record no. 022 and 0057).

### Eithics statement

All experiments were conducted in accordance with the European Community Council Directive 2010/63/EU for the Care and Use of Laboratory Animals of Sept. 22, 2010 (Chapter 1, Article 1 no.3) and National Journal of Law act of Jan. 15, 2015 for Protection of animals used for scientific or educational purposes (Chapter 1, Article 2 no.1). All methods involving zebrafish embryos/larvae were in compliance with ARRIVE guidelines. The works with genetically modified microorganisms were authorized by the Polish Ministry of the Environment (No. 179/2021).

### Cloning

For overexpression experiments, the full length mTurquoise cDNA was amplified by PCR from the p3E-p2a-mTurquoise plasmid (a gift from David Tobin; Addgene plasmid #135213) via the *Stu*I and *Xba*I sites of the pCS2 expression vector (a gift from Amro Hamdoun^48^; Addgene #34931) to generate pCS2-P2A-mTurquoise (control plasmid), as presented in the schematic (Fig. 4a). Then, the cDNA coding for full length Mcpip1 (XM_021466808**)** was amplified by PCR to introduce *EcoR*I and *EcoR*V and cloned into the *EcoR*I and *StuI* sites of the pCS2-P2A-mTurquoise plasmid to generate the Mcpip1 WT plasmid. To clone vectors containing sequences encoding catalytically inactive Mcpip1, site-directed mutagenesis of D112 into N112 was performed. The sequences of the primers used for cloning are listed in Supplementary Table S1.

### In vitro* transcription*

The pCS2 vectors (Control, Mcpip1 WT and Mcpip1 DN) were linearized with *Not*I digestion and cleaned using the PCR Mini Kit (Syngen). Then, the mRNAs were synthesized in vitro from the SP6 promoter using the mMESSAGE mMACHINE SP6 kit (Ambion AM1340) according to the manufacturer’s protocol. Transcribed mRNAs were purified using an RNA Clean & Concentrator kit (Zymo Research). A NanoDrop 2000 spectrophotometer (Thermo Fisher Scientific) was used to calculate the concentration of mRNA, which was then diluted to a concentration of 200 ng/µl. The integrity of mRNA was also confirmed by denaturing agarose electrophoresis.

### Microinjection of mRNA into fertilized zebrafish eggs

For overexpression experiments, 3.5 µl (700 ng) of each mRNA was mixed with 0.5 µl phenol red (Merck, P0290) and microinjected into the yolks of approximately 100 zebrafish eggs at the one-cell stage using a WPI Picopump PV820 microinjector (2 nl). The microinjected embryos were collected at 4 and 6 hpf for further RNA analysis.

### RNA isolation and quantitative PCR

For RNA isolation, ~ 10 zebrafish embryos were collected in fenozol (A&A Biotechnology), frozen and stored at -80 °C. Then, the embryos were homogenized using a homogenizer (OMNI International), and total RNA was extracted using the phenol–chloroform method. cDNA was synthesized using M-MLV reverse transcriptase (Promega), and quantitative real-time PCR was performed with SYBR Green Master Mix (A&A Biotechnology) and a QuantStudio3 thermocycler (Thermo Fisher Scientific). *Rps11*, *eef1*, *acbt2* and *rpl13a* were used as a reference genes^49,50^ The primer sequences are listed in Supplementary Table S1. To validate specificity of the qRT-PCR reaction, melt curve analysis was performed at the end of each assay. Agarose gel electrophoresis was performed to ensure presence of a single product of predicted length at the end of the qRT-PCR reaction (Supplementary Fig. S3d).

### RNA sequencing

The poly(A) mRNA fraction from total RNA was isolated with a Dynabeads mRNA DIRECT Micro Kit (Thermo). The sequencing library for each RNA sample was prepared according to the protocol provided by the manufacturer using the Ion Total RNA-Seq Kit v2 (Thermo). The libraries were generated from 1–15 ng of mRNA by fragmenting the mRNA with RNaseIII, purifying the fragmented RNA, and hybridizing and ligating it with Ion adaptors. Subsequently, the RNA products were reverse transcribed and amplified to double-stranded cDNA and then purified using a magnetic bead-based method. The molar concentration and size of each cDNA library were determined using the DNA HS Kit on a Bioanalyzer 2100 (Agilent). Each library was diluted to ~ 53 pM before template preparation. Up to three barcoded libraries were mixed in equal volume and used for automatic template preparation on the Ion Chef instrument (Thermo) using reagents from the Ion PI Hi-Q 200 Kit (Thermo) and Ion PI v3 Proton Chip. All samples were sequenced on the Ion Proton System (Thermo) according to the manufacturer’s instructions.

Signal processing and base calling were performed with Torrent Suite version 5.14.0. Raw reads were mapped to *D. rerio* Ensembl genome version GRCz11 using STAR (version 2.7.10a)^51^ and bowtie2 (version 2.4.4)^52^ for unmapped reads. Gene counts were created with htseq-count^53^ using the Ensembl gene model. Differential expression was analyzed with DESeq2 version (version 1.40.1). RNA sequencing data were deposited in the GEO repository (under accession no: GSE232220).

Functional annotation of DEGs (*fold change* > 1.5 and *p value* < 0.05) was performed using the R package ClusterProfiler version 4.4^54^. Gene lists were searched using the Entrez gene annotation (ENTREZ_GENE_ID), with the *D. rerio* background dataset used for analyses. Volcano plots and dot plots were created using the ggplot2 libraries in R.

### Imaging

Each stage of zebrafish embryo development was observed and photographed under an inverted microscope (Leica DMi1 under Flexacam C1). The fluorescence signal from mTurquoise-fused protein was observed under a fluorescence stereomicroscope (Zeiss Discovery V12 with a PentaFluar S filter slider equipped with a Zeiss Axiocam 705 mono camera). The excitation wavelength was 436 nm, and the emission wavelength was 480 nm.

### Statistics

All graphs were created using CorelDRAW 2021 (Corel Corporation), and all statistical analyses, including unpaired *t*-test and one-way ANOVA followed by Tukey`s multiple comparisons test, were performed using GraphPad Prism 8 (GraphPad Software).

### Supplementary Information

Supplementary Information.

## Data Availability

Sequencing data have been deposited in NCBI's Gene Expression Omnibus and are accessible through GEO Series accession number GSE232220. Any additional data are available from the corresponding author upon reasonable request.
