# Supplementary material for: MCPIP1 functions as a safeguard of early embryonic development
Source: Sci Rep. 2023 Oct 7;13:16944. doi: 10.1038/s41598-023-44294-1 (PMC10560294; doi:10.1038/s41598-023-44294-1)
Supplement: Supplementary file 1 — Supplementary Information. [file 41598_2023_44294_MOESM1_ESM.pdf]

Supplementary materials

Supplementary Table S1.

List of primer sequences used in this study.

**Sequences of primers used for cloning**		
**Name**	**Forward (5’->3’)**	**Reverse (5’->3’)**
P2A-mTurquoise (*Stu*I and *Xba*I)	ATAGGCCT GGAAGCGGAGCTACTAACTTCAG	ATTCTAGA TTACTTGTACAGCTCGTCCATGC
zc3h12a_CDS (*Eco*RI and *Eco*RV)	ATGAATTC TCCATCCGGCATCAATATGCAG	ATGATATC GAAGAACTCTCCGCTGGATTTGAG
Mcpip1_D112N	CCTATAGTTATTAATGGCAGCAATGTGG	CTTCAGGTCACTTTCAGCTTCAG
**Sequences of primers used for qRT-PCR**		
**Gene**	**Forward (5’->3’)**	**Reverse (5’->3’)**
foxh1	ATAAGTCCACAGGGATGGCG	ACAGAGTCCCTCCAGCCTTT
nrarpa	TTTTCTGCGAGAAGCCCCAT	CTGGAAACATCAGCAACGCC
rasl11b	CTAAGCGGTTTATCGGCGAC	GTGGTCAGTGCAGCTCAATC
rhov	GCTGATGGATACTGCTGGACA	TGTTGGGTTCACCACACTGAA
rps11	TAAGAAATGCCCCTTCACTG	GTCTCTTCTCAAAACGGTTG
zc3h12a	CCGAGGGCACCGAAATATCA	CATCAGGTGGCATGAACTTGTC
eef1	CTACCCTCCTCTTGGTCGCT	GGAACGGTGTGATTGAGGGAA
actb2	CATCCTCCGTCTGGACTTGG	CGCAAGATTCCATACCCAAGAA
rpl13a	TGGAGGACTGTAAGAGGTATGCT	GGACAACCATGCGCTTTCTC

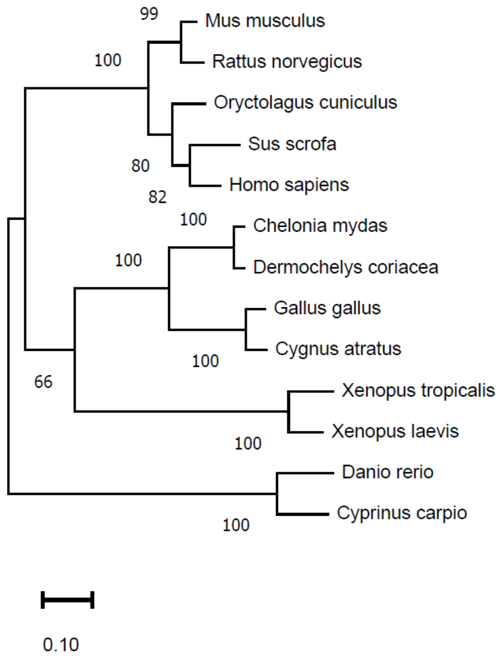

**[%]**	***D. rerio***	***C. carpio***	***X. tropicalis***	***X. laevis***	***C. mydas***	***D. coriacea***	***G. gallus***	***C. atratus***	***M. musculus***	***R. norvegicus***	***O. cuniculus***	***S. crofa***	***H. sapiens***
D. rerio	100												
C. carpio	82.90	100											
X. tropicalist	50.28	49.72	100										
X. laevis	51.20	51.43	86.72	100									
C. mydas	50.00	50.44	54.39	53.99	100								
D. coriacea	50.45	50.88	54.49	54.08	96.08	100							
G. gallus	50.72	51.17	53.44	54.00	76.93	76.28	100						
C. atratus	50.00	51.55	53.96	54.26	75.50	74.84	92.53	100					
M. musculus	50.19	50.37	54.34	54.70	59.76	59.86	59.30	59.58	100				
R. norvegicus	50.19	50.74	52.87	53.59	59.59	59.35	58.43	59.05	92.79	100			
O. cuniculus	51.58	51.01	53.79	53.78	60.07	60.51	58.93	58.84	83.56	82.72	100		
S. scrofa	49.44	49.45	54.03	54.53	61.69	62.15	61.03	61.37	81.00	81.87	84.36	100	
H. sapiens	50.28	50.46	53.05	53.41	60.07	59.83	58.58	58.67	82.55	82.89	86.31	86.25	100

Supplementary Figure S1. Phylogenetic trees based on the amino acid sequences of Mcpip1 of zebrafish (*D. rerio*) and selected representative species of other groups of vertebrates. For alignment, accession numbers are as follows: *D. rerio* - XP_021322483.1, *Cyprinus carpio* - XP_042596295.1, *Xenopus tropicalis* - XP_004911656.2, *X. laevis* - XP_018105042.1, *Chelonia mydas* - XP_043388107.1, *Dermochelys coriacea* - XP_038233907.1, *Gallus gallus* - XP_040545661.1, *Cygnus atratus* - XP_035417882.1, *Mus musculus* - NP_694799.1, *Rattus norvegicus* - NP_001071139.1, *Oryctolagus cuniculus* - XP_051688026.1, *Sus scrofa* - XP_020951625.1, *Homo sapiens* - NP_079355.2.

```
H.s. ZC3H12A[NP_079355.2]     --------------------------------------------------VVIDGSNVAMSHGNKEVFSCRGILLAVNWFLERGHTDITVFVPSWRKEQPRP 189
D.r. Zc3h12a[XP_021322483.1] --------------------------------------------------IVIDGSNVAMSHGNKEVFSCRGIELAVNYFLDRGHRNITVFVPSWRKEQPRP 160

H.s. ZC3H12B[NP_001010888.3] --------------------------------------------------VVIDGSNVAMSHGNKEEFSCRGIQLAVDWFLDKGHKDITVFVPAWRKEQSRP 244
D.r. Zc3h12b[NP_001164500.1] --------------------------------------------------IVIDGSNVAMSHGNKEVFSCLGIQLAVDWFLEKGHKDITVFVPAWRKEQSRP 216

H.s. N4BP1   [NP_694574.3]    --------------------------------------------------IVIDGSNVAITHGLKKFFSCRGIAIAVEYFWKLGNRNITVFVPQWRTR---R 668
D.r. N4bp1   [NP_956081.2]    --------------------------------------------------IIIDGSNVAMAHGLHRVFSCRGIAIAVEAFWRRGHREITVFVPQWRQK---K 649

H.s. KHNYN   [NP_056114.1]    ----------------------------------------------DLRHIVIDGSNVAMVHGLQHYFSSRGIAIAVQYFWDRGHRDITVFVPQWRFS---K 488
D.r. Khnyn   [XP_005166358.1] AKFSNTIPKAKPERGAVASVVTGPQRFLEGLKKPFSLQLSDQPGDAQLRHVIIDGSNVAMSHGLGVFFSCRGIALAVQHFWAEGHREIMVFVPQWRQK---N 636
                                                                                ::*******: **    **. ** :**: *   *: :* **** **

H.s. ZC3H12A[NP_079355.2]     DVPITDQHILRELEKKKILVFTPSRRVGGKRVVCYDDRFIVKLAYESDGIVVSNDTYRDLQGERQEWKRFIEERLLMYS----------------------   268
D.r. Zc3h12a[XP_021322483.1] DVPISDQHILGELERKKFLVFTPSRRVGGKRVVCYDDRFIVKLAHDLDGIIVSNDTYRDLQSERPEWKRFIEERLLMYS----------------------   239

H.s. ZC3H12B[NP_001010888.3] DAPITDQDILRKLEKEKILVFTPSRRVQGRRVVCYDDRFIVKLAFDSDGIIVSNDNYRDLQVEKPEWKKFIEERLLMYS----------------------   323
D.r. Zc3h12b[NP_001164500.1] DAPIKDQEILRKLEKEKILVFTPSRRVQGRRVVCYDDRFIVKLACDSDGIIVSNDNYRDLQNEKPEWKKFIEERLLMYS----------------------   295

H.s. N4BP1   [NP_694574.3]    DPNVTEQHFLTQLQELGILSLTPARMVFGERIASHDDRFLLHLADKTGGIIVTNDNFREFVNESVSWREIITKRLLQY-----------------------   746
D.r. N4bp1   [NP_956081.2]    DPNITEQHFLNQLENLRLLSFTPSREVCGHRISSHDDRFLLHLAEKTGGVIVTNDNLRDFVSQSEAWRRIIHERLLQF-----------------------   727

H.s. KHNYN   [NP_056114.1]    DAKVRESHFLQKLYSLSLLSLTPSRVMDGKRISSYDDRFMVKLAEETDGIIVSNDQFRDLAEESEKWMAIIRERLLPFTFVGNLFMVPDDPLGRNGPTLDE   589
D.r. Khnyn   [XP_005166358.1] NSKIKEKHYLNELHDLGLLSYTPSREVEGKRIISYDDRFMLDLAQKTNGVIVTNDNLRDLVDESPAWRDIIKKSLLQYVFAGDQFMLPDDPLGRGGPHLRD   690
                              :  : :.. * :*    :*  **:* : *.*: .:****::.** . .*::*:**  *::  :   *  :* : ** :
```

[%]	***H.s.* ZC3H12A**	***D.r.* Zc3h12a**	***H.s.* ZC3H12B**	***D.r.* Zc3h12b**	***H.s.* N4BP1**	***D.r.* N4bp1**	***H.s.* KHNYN**	***D.r.* Khnyn**
***H.s.* ZC3H12A**	100							
***D.r.* Zc3h12a**	87.79	100						
***H.s.* ZC3H12B**	83.21	80.15	100					
***D.r.* Zc3h12b**	83.21	80.15	93.89	100				
***H.s.* N4BP1**	48.82	51.18	49.61	49.61	100			
***D.r.* N4bp1**	52.76	55.12	51.18	51.18	70.08	100		
***H.s.* KHNYN**	54.69	57.03	54.69	53.91	59.84	63.78	100	
***D.r.* Khnyn**	50.78	52.34	50.78	50.00	60.63	68.50	59.74	100

Supplementary Figure S2. Alignment of the PIN domain amino acid sequences of human and zebrafish PIN-domain-like superfamily members between *Homo sapiens (H.s.)* and *Danio rerio (D.r.)*. Asterisk (*) denotes an identical residue, a colon (:) denotes conserved substitutions, and a period (.) denotes semiconserved substitutions.

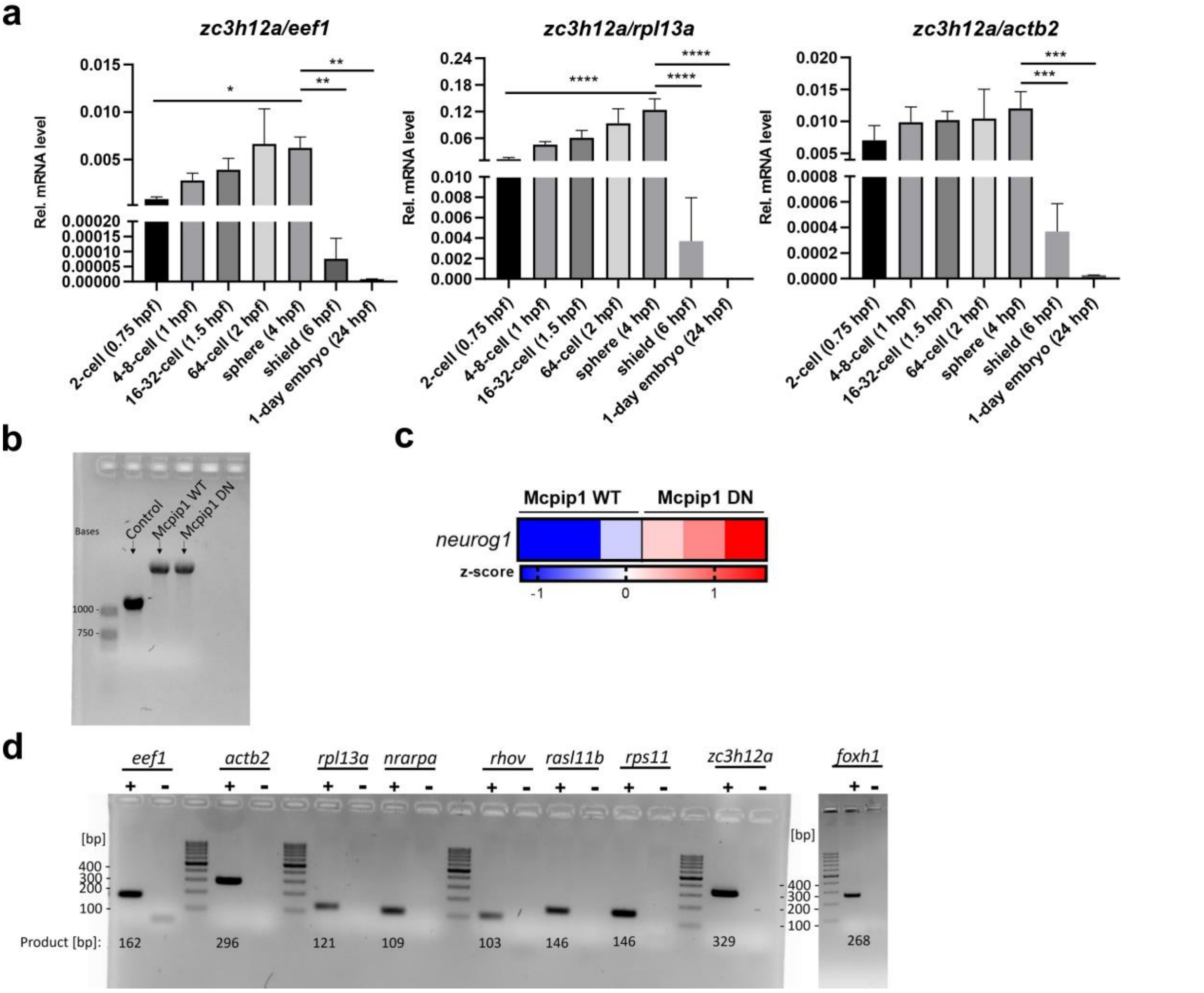

Supplementary Figure S3. a. Real-time PCR analysis of *zc3h12a* mRNA level. *Eef1, rpl13a* and *actb2* were used as a reference genes. Data represent the mean ± SD, **p* < 0.05, ***p* < 0.01, ****p* < 0.001, *****p* < 0.0001 by one-way ANOVA (only selected comparisons are shown). **b.** RNA denaturing agarose gel electrophoresis of ~300ng synthesized mRNA. The Low Range ssRNA Ladder (New England BioLabs) was used. **c.** Heatmap presenting normalized (z-score) *neurog1* mRNA level with p *value < 0.1,* based on RNA-seq data. **d.** DNA agarose gel electrophoresis of QRT-PCR products. Plus (+) means cDNA and minus (-) means water as a template. The 100 bp GeneRuler Ladder (New England BioLabs) was used.
